# Presenting Your Best Self(ie): The Influence of Gender on Vertical Orientation of Selfies on Tinder

**DOI:** 10.3389/fpsyg.2017.00604

**Published:** 2017-04-21

**Authors:** Jennifer R. Sedgewick, Meghan E. Flath, Lorin J. Elias

**Affiliations:** Department of Psychology, University of SaskatchewanSaskatoon, SK, Canada

**Keywords:** selfies, posing, height preference, sexual dimorphism, power, grounded cognition, attraction, online dating

## Abstract

When taking a self-portrait or “selfie” to display in an online dating profile, individuals may intuitively manipulate the vertical camera angle to embody how they want to be perceived by the opposite sex. Concepts from evolutionary psychology and grounded cognition suggest that this manipulation can provide cues of physical height and impressions of power to the viewer which are qualities found to influence mate-selection. We predicted that men would orient selfies more often from below to appear taller (i.e., more powerful) than the viewer, and women, from an above perspective to appear shorter (i.e., less powerful). A content analysis was conducted which coded the vertical orientation of 557 selfies from profile pictures on the popular mobile dating application, Tinder. In general, selfies were commonly used by both men (54%) and women (90%). Consistent with our predictions, a gender difference emerged; men's selfies were angled significantly more often from below, whereas women's were angled more often from above. Our findings suggest that selfies presented in a mate-attraction context are intuitively or perhaps consciously selected to adhere to ideal mate qualities. Further discussion proposes that biological or individual differences may also facilitate vertical compositions of selfies.

## Introduction

When creating an online dating account, choosing a profile photo becomes a thoughtful process (Ellison et al., 2006), as it can predict the success of initiated contact (Hitsch et al., 2006). This is particularly true for the most currently used mobile dating application, Tinder (SurveyMonkey Intelligence, 2016), due to its emphasis on the profile photo; that is, its format promotes users to make rapid judgements based on physical attractiveness, a primary determinant in the early stages of mate selection (Li et al., 2013; Fletcher et al., 2014).

Curiously, mobile camera self-portraits or “selfies” are often used for the profile's main image; 57% of men and 90% of women from our sample of Tinder users chose this method of representation. By considering evolutionary theories of attraction and grounded cognition, how the selfie-taker vertically orients the camera may be from an angle which perceptually manipulates qualities that are attractive to the opposite sex (e.g., height, perceptions of power). Specifically, the current study explores how heterosexual men and women vertically portray themselves relative to the viewer—from above, or from below—for selfies displayed on Tinder.

Attraction to vertical cues of physical height are largely suggested to emerge from humans' sexual size dimorphism. Males, on average, tend to exceed females in height (Gray and Wolfe, 1980; Ruff, 2002), thereby masculinity is strongly associated with the expression of tallness (Jackson and Ervin, 1992). However, a sizeable stature may also serve as a physical cue for females to other evolutionary advantages such as dominance, social status, and the ability to attain resources (Buss, 1989, 1994; Fiske, 2004). Women from Western cultures consistently demonstrate this attraction to tall men as reported from surveys of ideal mate characteristics (Pierce, 1996; Courtiol et al., 2010; Yancey and Emerson, 2014). Furthermore, the male-taller norm is evident from investigations of actual height differences between couples (Gillis and Avis, 1980). Women's robust height preference illuminates why taller males tend to report more sexual partners (Frederick and Jenkins, 2015) and reproductive success (Pawlowski et al., 2000; Nettle, 2002a) than their shorter-statured counterparts.

The literature on height preferences of men seeking women demonstrate a less stringent ideal, as men report a significantly weaker preference for respectively shorter women (Pawlowski, 2003; Fink et al., 2007). Research examining real-life data of online dating behavior revealed that men made first-contact emails to women of *average* height 43% more than women taller than 6′3, whereas women initiated contact with men of *above*-average height 65% more than shorter men (Hitsch et al., 2006). The preference for average height similarly corresponds to the stature of women with the most reproductive success (Nettle, 2002b), though this success is comparatively lower than that of taller men (Nettle, 2002a). The decreased importance of women's height is perhaps surprising given that men value external qualities for potential mates more so than women (Regan et al., 2000; Olivola et al., 2009). However, height is an attribute unrelated to female fertility (Nettle, 2002b), effectively decreasing this cue to represent any evolutionary advantage.

Although physical height is a significant feature of mate selection, this cue is absent from Tinder's profile layout unless explicitly stated by the user in their profile's tagline. Alternatively, the profile photo may be spatially manipulated to emulate the appearance of height either by orienting the camera from above or below the vertical axis, thereby exploiting the perception of the viewer to appear taller or shorter than the photographic subject. Research examining the effect of facial head-tilt on judgments of gender have found that pictures of faces with an upwards head-tilt, thus being perceived from below, are perceived to be more masculine, and faces tilted-downwards, so from an above perspective for the viewer, as more feminine (Main et al., 2010). These directionalities of head-tilt are parallel to ratings of facial attractiveness (Burke and Sulikowski, 2010; Sulikowski et al., 2015). Habitually learned perceptions of faces arising from height differences are proposed to guide these perceptions (DeBruine et al., 2006), though a complementary theory is proposed from the area of embodied cognition.

Grounded theories of cognition pioneered by Lakoff and Johnson (1980, 1999) posit that abstract concepts, such as power, are mentally associated with vertical spatial orientations (i.e., up is perceived as powerful and down, powerless; Barsalou, 1999). This association is exhibited by the English language, whereby common idioms of power and submission are vertically positioned: one has control *over* someone or be *under* their control, *rise* or *fall* from power, or be of *high* ranking or the *low* man on the totem pole (Lakoff and Johnson, 1980, p. 16). Considering this knowledge, Meier and Dionne (2009) predicted that the attractiveness of men's and women's portraits would depend on their spatial congruency with power; specifically, males are a proxy for “up” due to masculine trait preferences related to power (i.e., dominance, high social status) and for females, a lack of power (i.e., faithfulness) corresponding with “down.” As predicted, men rated women's portraits as more attractive when *identical* photos were presented at the bottom of a computer screen (vs. top), whereas women were more attracted to images of men at the top of the screen (vs. bottom).

The directionality of the power metaphor with gender suggests a clear parallel with the literature from evolutionary psychology; “up” or tallness is signified with masculinity, and “down” or being shorter indicates femininity. However, as previously stated, height is not always an available cue in an online dating environment. Therefore, we propose that when choosing the focal point of the profile—the first profile picture—individuals may intuitively know to select an image where the vertical angle of the camera is consistent with how they want to be presented to the opposite sex: for men, from below to appear larger and dominant (i.e., powerful), and for women, from above to look smaller and submissive (i.e., less powerful). Due to the control from the self-display of the smartphone's frontal camera, an individual can easily manipulate this angle by taking a selfie, thus *appearing* taller or shorter relative to the viewer. We chose to explore strictly selfies for this reason, and because of the increased likelihood that the selfie was taken explicitly to portray attractiveness.

The purpose of the current study is to compare the vertical spatial orientation of men's and women's selfie profile pictures from Tinder, to which we predict that men will more often choose selfies oriented from below (vs. above), and women will depict selfies more often from above (vs. below). The current study will contribute to research on human attraction by exploring if physical preferences reported from previous studies are embodied by individuals in a realistic mate-attraction setting. Further, the study will inform how men and women represent a vertical orientation for selfies, a contrast to the lateral exploration of this media phenomenon (Bruno et al., 2015, 2016; Lindell, 2015).

## Methods

### Sampling

A total of 962 profile photos were collected from Tinder. From this total were 508 profiles of women ranging from 18 to 44 years of age (*M* = 24.43, *SD* = 4.7), and 454 profiles of men between the ages of 18–56 (*M* = 30.5, *SD* = 8.39). Standard selfies—informal self-portraits portraying only the selfie-taker (Bruno et al., 2015)—were then parsed from the total. Mirror-selfies were also excluded (26 men, 13 women), because altering the vertical camera position does not affect the relative perspective of the model to the same effect as non-mirror selfies. Our final data set comprised of 665 selfies, whereby 247 were from men's profiles and 457 were from women's. Selfies accounted for 54% of men's and 90% of women's profile pictures.

As previously specified, Tinder was an ideal online dating platform due to its current popularity and because of the layout's emphasis on the profile photo; only the first name, age, name of employer, and one picture is displayed as users “swipe” to explore Tinder profiles. Thus, the decision to “swipe right,” or approve permission of contact by another user is largely founded by physical appearance, as Tinder only presents profiles of users specified from the account's search features (i.e., gender, age range, proximity in kilometers). Collection of the images are compliant with Tinder's privacy policy (Tinder Inc and Privacy Policy, 2016). Analysis of this collection was not subject to review by the University of Saskatchewan's Research Ethics Board (REB); the Standard Operating Procedures from our Human Ethics Policies states that data derived from observing publicly available media does not require REB review provided that no individuals' information may be identified (Research Ethics Office, 2012).

For the study, two Tinder profiles were created—one of a man seeking women, and one of a woman seeking men. To access enough profiles for a sufficient data set, the “Discovery Settings” were set to include Tinder users over the age of 18, within 160 km from the University of Saskatchewan campus, and toward the opposing gender of our profile's user. At that point we could view the profiles of each targeted gender, to which we coded profile images until there were no other users available within our demographic interests. Images were collected on May 10th, 2016.

### Coding

The vertical orientations of the models within the selfie sample set were coded by six research assistants (three males, three females) blind to the hypotheses of the study. Our rationale for assessing selfies' vertical orientation using human scoring rather than an objective measurement was motivated by two factors: (1) to understand how individuals experience the portrait's subject relative to themselves, and (2) because of the inability of Facial Recognition Software to detect the degree of head-tilt due to obscure photographic compositions, poor image resolution, or occluded views of the face (e.g., hair, sunglasses).

Assistants were seated at eye-level to a desktop computer and presented with the following instructions:

“*Please say which vertical location you think you are relative to the person in the picture—above them, below them, or if they are at an equal level to you”*

**Figure 1:**
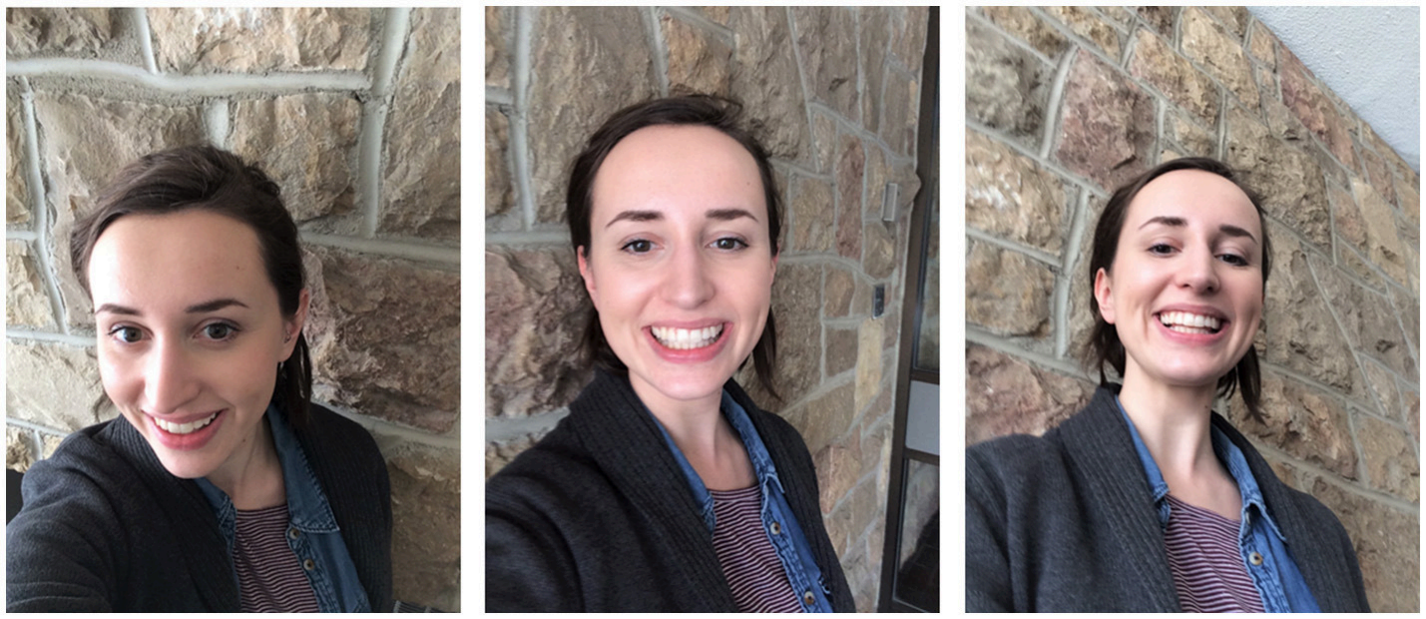
**Examples of vertical camera angle manipulation**. From left to right, the presented images illustrate selfies photographed from an above, frontal, and below perspective. The portraits are modeled by a research assistant to maintain confidentiality of the sampled Tinder users.

To decrease the coding time from the large sample set, assistants verbally indicated their relative spatial judgment for each photo while the primary researcher coded their selection on a separate computer. Poses oriented from above were coded as +1, poses from below as −1, and a straight pose as 0 (i.e., no obvious head-tilt; see Figure 1 for examples of each pose).

The posing choices for all assistants were then compiled in a spreadsheet for further comparison. The directionality of portrait orientation for each selfie was determined to be from above, below, or equal if there was agreement among four of the six raters. Images with less than four agreements were discarded prior to analysis; this equated to 95 images (14%) and with a moderate inter-rater agreement (Altman, 1999) determined using Cohen's Kappa, κ = 0.4, (95% CI, 0.035–0.044), *p* < 0.001. Our choice to only include images with at least four agreements was due to the large number of central posing choices reported by assistants on images with an otherwise adequately reported directional bias.

## Results

**Figure 2:**
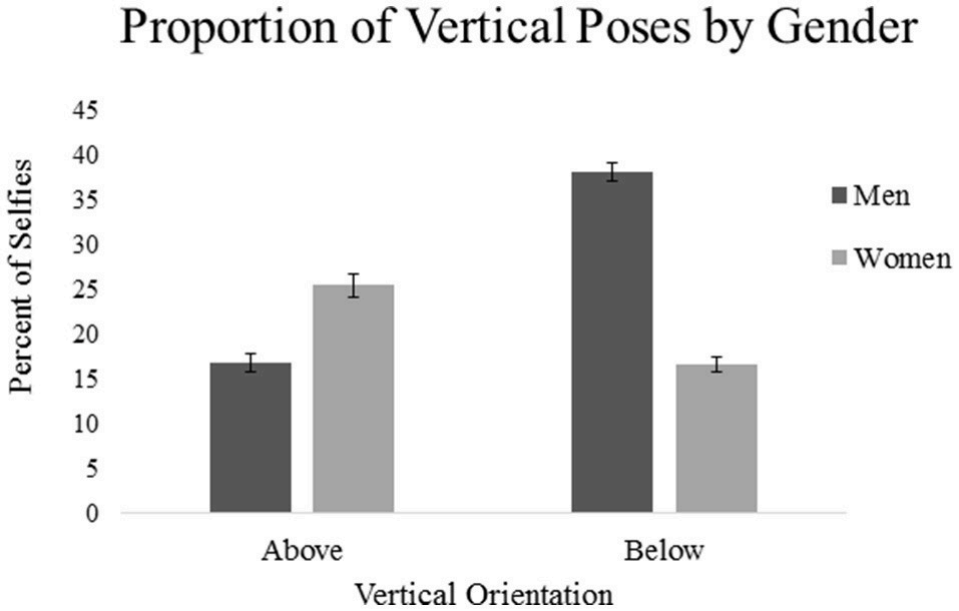
**Proportion of vertical poses (±SE) based on gender**. The figure illustrates the proportional difference between men and women's tendency of taking vertical selfies; that is, when excluding neutral poses, men displayed a bias for portraits of selfies from below, whereas women alternatively presented an above-bias.

Frequencies of the spatial orientation from the selfie sample suggests that distinctly vertical compositions of the camera were commonly used by both men and women, as profile photos with an above or below orientation were presented in 55.1% and 42.1% of pictures, respectively (see Table 1 for all spatial frequencies). To determine if there was a difference between posing orientation depending on gender, a one-way ANOVA was conducted. However, the ANOVA's homogeneity of variance assumption was violated as indicated by the Levene's test, *F*_(1, 554)_ = 13.55, *p* < 0.001; alternatively, a Welch's ANOVA was used. A significant difference between groups was revealed, *F*_(1, 398.4)_ = 24.94, *p* < 0.001, η^2^ = 0.03, demonstrating that men oriented the camera more often from below (*M* = −0.213, *SD* = 0.644) than women (*M* = 0.089, *SD* = 0.644) in selfies presented on Tinder (see Figure 2 for proportional differences).

**Table 1:** **Frequency and percentage of posing**.

**Gender**	**Above**	**Below**	**Frontal**
**FREQUENCY/PROPORTION**			
Men	35 (16.9%)	79 (38.2%)	93 (44.9%)
Women	58 (25.5%)	89 (16.6%)	202 (57.9%)
Total	93 (100%)	168 (100%)	295 (100%)

To examine if the directionality of men's and women's poses were significantly different from zero (i.e., a straight pose), two one-sample *t*-tests were computed. The analyses corresponded with our predictions; men oriented the camera more often from below, *t*_(206)_ = −4.291, *p* < 0.001, Cohen's *d* = 0.598, and women, more often from above, *t*_(348)_ = 2.577, *p* = 0.01, Cohen's *d* = 0.276. Taken together, the results illustrate the contrast between how men and women choose to spatially represent themselves in a mate-attraction context.

## Discussion

Selfies exhibited in online dating profile photos were predicted to vary by vertical camera angle depending on the sex of the individual. Our results revealed that profile photos of men and women users of the mobile application, Tinder, exhibited opposing vertical biases; the camera's perspective was presented more often from below for men, and above for women. These findings simultaneously demonstrate a mechanical bias of selfies within a mate attraction context, as profile photos were not only chosen, but also taken by the Tinder user.

An effect of manipulating a selfie's vertical spatial dimension is that it creates the illusion of a height disparity between the model and the viewer. The findings of the current study suggest that individuals are intuitively or perhaps consciously aware of this phenomenon, as the composition of profile photos were consistent with the height ideals of the opposite sex. Specifically, men with selfies oriented from below facilitate the perception of tallness, a feature robustly reported from women's mate preferences (Pierce, 1996; Courtiol et al., 2010; Yancey and Emerson, 2014). By contrast, women's prevalence of selfies taken from overhead conveys relative shortness to the viewer, a smaller yet significant height preference reported by men (Pawlowski, 2003; Fink et al., 2007).

Emphasizing this sexual dimorphism (Gray and Wolfe, 1980; Ruff, 2002) may serve to activate assumptions of features that are evolutionarily attractive to the opposite sex. As reported from cross-cultural research by Buss (1989, 1994), tall men are commonly associated with protection, high social status, and access to resources, whereas shorter women are perceived to symbolize faithfulness and subordination. A comparable theory comes from the area of grounded cognition, though its emphasis on verticality is its link to perceptions of power (Barsalou, 1999). This association derives from the phenomenon proposed by Lakoff and Johnson (1980, 1999) that vertical space is a proxy for power due to its mental representation—powerful is up, less powerful is down (Schubert, 2005). Due to average height differences, men physically tower over women, therefore alluding to a perceived power differential. Research has found this metaphorical transfer to influence attraction; Meier and Dionne (2009) demonstrated that men rated women's portraits as more attractive when presented at the bottom of a computer screen (vs. top), whereas the alternative was found for women viewing men's portraits. Although attractiveness was predicted by its spatial presentation rather than height, we propose that grounded theory is a complementary explanation rather than a central one due to the extensive evidence on height preferences and mate selection.

In addition to manipulating height preferences, we speculate that other physical features related to men's and women's attractiveness can be enhanced by a selfie's camera angle. For men, a broad jawline is a sexual dimorphism (Weston et al., 2007) that is similarly referenced to masculinity. Facial-width has been found to correlate with both perceptions (Alrajih and Ward, 2014; Mileva et al., 2014) and self-reported (Lefevre et al., 2014) dominance, and is a physical preference considered by women for short-term relationships (Valentine et al., 2014). Taking a picture from below thus serves the purpose of creating an illusion of a pronounced jaw, as it obscures the size of the jaw relative to the face. By contrast, women may choose to take a photo from above to distort the head in relation to body size, accordingly deemphasizing a feature commonly misrepresented by women—their weight (Engstrom et al., 2003; Toma et al., 2008). An above camera angle would therefore reduce not only the perceived physical height of the woman, but also to flatter their physical frame. Aside from the conscious effort for women to conform to contemporary trends of body ideals, capturing a physically appealing figure can also implicitly signal fertility health (Jungheim et al., 2013), a biological advantage which is more strongly linked to reproductive success for females than height (Nettle, 2002b).

For the current study, subjective measurements of vertical camera angle were ideal to validate how individuals perceived themselves relative to the portrait's model (i.e., taller than or shorter than the model). Consequently, the vertical orientation was only possible to be categorically quantified (i.e., above, below, or central) as opposed to a continuous variable (i.e., degree of vertical angle measured). A resulting trade-off is that we could not compare the extent of vertical exaggeration, only its distinct directionality. An additional short-coming was that unreliable agreements of selfie-composition between raters led to 14% of discarded data, a consequence that could have been eliminated from an objective measurement (e.g., facial-analysis software). Upon inspection of the discarded stimuli, however, it is possible that the variability of posing choices may be due to assistants' sensitivity to report a neutral rather than directional pose, as the data points failed to meet our selected agreement standard often displayed an even split between one vertical directionality and a central pose. The results of the current study therefore capture humanly perceivable differences rather than small deviations of camera angle.

An additional limitation is from the nature of using a content analysis, which is that we have restricted access to fruitful information of the Tinder users. A variable of interest for future examination is the photographic experience of the selfie-taker. Individuals with knowledge of photographic techniques may take more selfies from above, as this perspective is considered as a more flattering presentation of a face (Phillips, 2006). This knowledge may be similarly learned through experience taking selfies. Research has shown that women upload selfies to photo-sharing applications such as Instagram (Sorokowska et al., 2016) more often than men. Women's increased experience taking selfies may be an additive variable for their predominant use of the above camera-tilt.

Future direction should also explore how individual differences modulate the vertical position of selfies. Conformity to stereotypical gender roles may predict the ideal portrayal of oneself, such that those with higher conformity may choose to exhibit themselves as taller (more masculine) or shorter (more feminine). Research by Bogaert and McCreary (2011) found that men with higher conformity to masculine norms conveyed a larger disparity between their self-reported and actual height. Men's gender conformity is also found to negatively correlate to the height of ideal female partners (Swami et al., 2008). The literature regarding gender norms and height for women is negligible, further insinuating the importance of men's height. However, women who desire to conform to perceived societal norms are more likely to misreport their weight (Larson, 2000). If our hypothesis regarding selfies as a means of deemphasizing weight is truthful, conformity to gender roles may also act as a modulating variable.

In conclusion, the present study provides novel insight on how human mate preferences correspond to mate-attraction behaviors. Our research demonstrates that when taking a selfie for presentation in a mate-attraction context, individuals choose to spatially orient themselves in a manner that is congruent with the opposing sex's height preferences; that is, from below to appear taller for men, and from above to portray relative shortness for women. This phenomenon may arise due to individuals initiating consciously-known selective cues of attraction, or from individual differences that warrant further exploration. The current findings contribute to a greater understanding of how evolutionary and conceptually grounded mechanisms can facilitate behavior in modern dating strategies and for capturing techniques of modern self-portraiture.

## Author contributions

JS designed the study. JS and MF conducted research. JS analyzed data. JS and LE wrote the manuscript.

### Conflict of interest statement

The authors declare that the research was conducted in the absence of any commercial or financial relationships that could be construed as a potential conflict of interest.
